# Clinical application of mesenchymal stem cell in regenerative medicine: a narrative review

**DOI:** 10.1186/s13287-022-03054-0

**Published:** 2022-07-28

**Authors:** Ria Margiana, Alexander Markov, Angelina O. Zekiy, Mohammed Ubaid Hamza, Khalid A. Al-Dabbagh, Sura Hasan Al-Zubaidi, Noora M. Hameed, Irshad Ahmad, R. Sivaraman, Hamzah H. Kzar, Moaed E. Al-Gazally, Yasser Fakri Mustafa, Homayoon Siahmansouri

**Affiliations:** 1 Department of Anatomy, Faculty of Medicine, Universitas Indonesia, Jakarta, Indonesia; 2 Master’s Programme Biomedical Sciences, Faculty of Medicine, Universitas Indonesia, Jakarta, Indonesia; 3 Dr. Soetomo General Academic Hospital, Surabaya, Indonesia; 4 Tyumen State Medical University, Tyumen, Russian Federation; 5 Tyumen Industrial University, Tyumen, Russian Federation; 6 Department of Prosthetic Dentistry, I.M. Sechenov First Moscow State Medical University (Sechenov University), Moscow, Russia; 7 Medical Technical College, Al-Farahidi University, Baghdad, Iraq; 8 Department of Dentistry, Al-Hadba University College, Mosul, Iraq; 9 Anesthesia Techniques Department, Al-Mustaqbal University College, Babylon, Iraq; 10 Anesthesia Techniques, Al–Nisour University College, Baghdad, Iraq; 11 Department of Medical Rehabilitation Sciences, College of Applied Medical Sciences, King Khalid University, Abha, Saudi Arabia; 12 Department of Mathematics, Dwaraka Doss Goverdhan Doss Vaishnav College, Arumbakkam, University of Madras, Chennai, India; 13 Veterinary Medicine College, Al-Qasim Green University, Al-Qasim, Iraq; 14 College of Medicine, University of Al-Ameed, Karbala, Iraq; 15 Department of Pharmaceutical Chemistry, College of Pharmacy, University of Mosul, Mosul, 41001 Iraq; 16 Department of Immunology, Faculty of Medicine, Tabriz University of Medical Sciences, Tabriz, Iran

**Keywords:** Mesenchymal stromal cell, Regenerative medicine, Stem cell therapy, Tissue regeneration, Cell therapy

## Abstract

The multipotency property of mesenchymal stem cells (MSCs) has attained worldwide consideration because of their immense potential for immunomodulation and their therapeutic function in tissue regeneration. MSCs can migrate to tissue injury areas to contribute to immune modulation, secrete anti-inflammatory cytokines and hide themselves from the immune system. Certainly, various investigations have revealed anti-inflammatory, anti-aging, reconstruction, and wound healing potentials of MSCs in many in vitro and in vivo models. Moreover, current progresses in the field of MSCs biology have facilitated the progress of particular guidelines and quality control approaches, which eventually lead to clinical application of MSCs. In this literature, we provided a brief overview of immunoregulatory characteristics and immunosuppressive activities of MSCs. In addition, we discussed the enhancement, utilization, and therapeutic responses of MSCs in neural, liver, kidney, bone, heart diseases, and wound healing.

## Introduction

In the last decade, stem cells are increasingly applied as a therapeutic method for numerous disorders. Stem cell therapy, traditionally applied for hematopoietic disorders, nonetheless, is now established for the treatment of non-hematologic disorders [1, 2].

Accumulating evidence has shown that mesenchymal stem cells (MSCs) offer an encouraging option for cell treatment and reconstruction of human tissues because of their differentiation multipotency, self‐renewal capacity, long‐term ex vivo proliferation, paracrine potentials, and immunoregulatory effect [3]. Furthermore, MSCs have the capability to support the progression and differentiation of other stem cells. They can release bioactive molecules, which is a key benefit in tissue regeneration [4, 5]. These properties result in progression of treatments for a wide range of diseases, such as diseases affecting the bone, neuron, lung, liver, heart, kidney, etc. [4]. Due to these features, it is obvious that MSCs will hold a major therapeutic role in clinical trials. Because of these properties, we provided a general overview of the latest trials that studied the effectiveness of MSCs in several diseases such as neural, liver, kidney, bone, heart diseases, and wound healing.

## Stem cells in regenerative medicine

In the last years, numerous studies have demonstrated that cellular therapy has exhibited great development in both in vitro and in vivo researches. Stem cells have the capability to self-renew, and also to differentiate into all cell types and are involved in physiological regeneration [6]. There are multiple stem cell sources of adult and pluripotent stem cells (PSCs) such as embryonic stem cells (ESCs) and induced pluripotent stem cells (iPSCs) for tissue regeneration. PSCs have a high potential for pluripotency and self-renewal, which makes these cells an important option for treatment of diseases. However, there are ethical issues when using these cells, in which ESCs are separated from blastocyst-stage embryos, requiring destruction of the embryo [7–9]. The results of studies have revealed the regenerative ability of iPSCs in preclinical setting and conducted the first clinical study for treatment of age-associated with macular deterioration [10, 11]. Nonetheless, the tumorigenicity risk remains unsolved. Because of these limitations, researchers began to investigate adult stem cells, the multipotent stem cells found in tissues and organs of adults. Various investigations have reported that stem cell therapy can regenerate and repair injured organs in vivo, including bone repair, cutaneous wound, pulpitis, and ischemic cardiac tissue through stem cell differentiation and production of new particular cells [12–15]. Moreover, some investigations have demonstrated that cultured adult stem cells release many molecular factors with anti-apoptotic, immunoregulatory, angiogenic, and chemoattractant features that stimulate regeneration [16–18]. Hematopoietic stem cells (HSCs) and MSCs are part of adult stem cells, which are the most widely used, generally because they can be isolated from individuals in diseased conditions.

## Mesenchymal stem cell

In the late 1960s, Friedenstein and colleagues discovered MSCs as multipotent stem cells for the first time [19]. MSCs are non-hematopoietic cells and have the capability to differentiate into various lineage including mesodermal (adipocytes, osteocytes, and chondrocytes), ectodermal (neurocytes), and endodermal lineage (hepatocytes) [20, 21]. At the beginning, it was thought that MSCs are “stromal” cells instead of stem cells [22]. Several investigators tried to alter the name of MSCs to medicinal signaling cells due to their function in secretion of some metabolites molecules in the sites of diseases, injuries, and inflammations [23, 24]. After that, some studies have stated that MSCs can release prostaglandin E2 (PGE2), which plays a major role in the self-renewal ability, immunomodulation of MSCs, and generating a cascade of events, that demonstrates the stemness of MSCs [25]. Therefore, the term mesenchymal stem cells is justified.

MSCs chiefly found in the bone marrow (BM) possess the ability of self-renewal and also display multilineage differentiation [8, 26, 27]. They were obtained from various tissues and organs including BM, adipose tissue, Wharton’s jelly, peripheral blood, umbilical cord, placenta, amniotic fluid, and dental pulp [3, 28–30]. MSCs can express a wide range of surface markers and cytokine profiles according to the origin of isolation [31]. Nevertheless, the common characterization markers of MSCs are CD73, CD105, CD90 and lacking expression of CD45, CD34, CD14 or CD11b, CD79α or CD19, and HLA-DR [32–34]. During the last decades, MSCs have shown various biological roles such as multilineage differentiation, immunomodulation, angiogenesis, anti-apoptotic and anti-fibrotic activity, chemo-attraction, and tissue repair development [35–37]. The MSCs have broad properties that make them a suitable source for cell therapy, such as stemness potency, easily isolation from different sources, they can be rapidly expanded in a large scale for clinical use, have less ethical issues as compared to ESCs, unlike iPSCs, MSCs transport a lower risk of teratoma formation, and they are beneficial for a wide scale of therapeutic applications due to their capability to migrate to injured tissue through chemo-attraction [38–40]. In addition, MSCs can release a variety of bioactive components including proteins, growth factors chemokines, microRNAs (miRNAs), and cytokines which can suggest their acceptable application [41].

## The biological roles of MSCs

MSCs have the ability to inhibit the immune response in inflammatory cytokine-rich situations, including infections, wounds, or immune-mediated disorders. These immunomodulatory properties were discovered in preclinical and clinical trials, where MSCs effectively suppressed T cell activation and proliferation along with stimulation of macrophages shift from M1 to M2 [42–44]. This specific performance of MSCs in the presence and absence of inflammatory mediators is termed MSC polarization. MSCs have the ability to migrate to damaged areas after systemic infusion and consequently exert a beneficial effect by various mechanisms, chiefly immunoregulation, and angiogenesis [45, 46]. Although the related mechanism-mediated MSC immunosuppression has not been entirely clear, it appears that cellular interaction, accompanied by many factors, performs the principal function in this process. In the presence of high levels of inflammatory cytokines, e.g., TNF-α and IFN-γ, MSCs release several cytokines including TGF-β and hepatocyte growth factor (HGF) and produce soluble factors including indoleamine 2,3-dioxygenase (IDO), PGE2, and nitric oxide (NO). These mediators suppress T effector cells and enhance the expression of FOXP3, CTLA4, and GITR in regulatory T cells (Tregs) to increase their immunomodulation effects [47–49]. Moreover, cell-to-cell communication facilitates the stimulation of Tregs by cytokine-primed MSCs [50]. Overexpression of inducible co-stimulator ligands (ICOSL) induces the stimulation of efficient Tregs [51].

In addition, MSCs can enhance the generation of Treg cells indirectly. According to the literature, MSCs stimulate M2 macrophage and alter the phenotype through secretion of extracellular vesicles in an in vitro study [52]. Also, M2 cells that are activated by MSCs express CCL-18 and induce Treg cells [53]. Moreover, MSCs increase the expression of cyclooxygenase 2 (COX2) and IDO, resulting in expression of CD206 and CD163 in M2 cells, as well as enhance the expression of IL-6 and IL-10 in the microenvironment [54]. The overexpression of IL-10 that is produced by dendritic cells (DCs) and M2 cells upon MSCs co-culture leads to further immunomodulation via inhibition of effector T cells [55, 56]. Furthermore, the secretion of IDO from MSCs can induce the proliferation, activation, and IgG releasing of B cells, thereby suppressing T effector cells [57, 58].

One of the typical properties of MSCs is their multipotency capacity in which these stem cells are able to differentiate into a number of tissues in vitro [59]. Chondrogenic differentiation of MSCs in vitro occurs commonly via culturing them in the existence of TGF-β1 or TGF-β3, IGF-1, FGF-2, or BMP-2 [60–63]. MSC differentiation into chondroblasts is characterized by the increasing of various genes such as collagen type II, IX, aggrecan, and proliferation of chondroblast cell morphology. During the process of chondrogenesis, FGF-2 promotes the MSCs induced with TGF-β1 or TGF-β3 and/ or IGF-1 [64]. According to the literature works, several molecular pathways such as hedgehog, Wnt/β-catenin, TGF-βs, BMPs, and FGFs can regulate chondrogenesis [65]. In addition, MSCs can exert the osteogenesis function by inducing MSCs with ascorbic acid, β-glycerophosphate, vitamin D3, and/or BMP-2, BMP-4, BMP-6, and BMP-7 [66].

One of the major abilities of MSCs is anti-fibrotic activity. These cells can differentiate into various cell lineages such as hepatocytes, both in vivo and in vitro [67]. MSCs contain multiple trophic factors which induce cells and matrix remodeling to stimulate progenitor cells and the recovery of damaged cells. MSCs can decrease myofibroblasts and reverse the fibrotic activity of injured tissues [68]. Furthermore, these cells release pro-angiogenic factors including VEGF, IGF-1, and anti-inflammatory factors that participate in the recovery of tissue function. For instance, MSCs can increase neovascularization of ischemic myocardium through VEGF in a mice model of heart disease [69]; also, IGF-1 exerts an advantageous effect on the survival and proliferation of cardiomyocytes [70].

## Bone marrow mesenchymal stem cell-based regenerative medicine

**Fig. 1:**
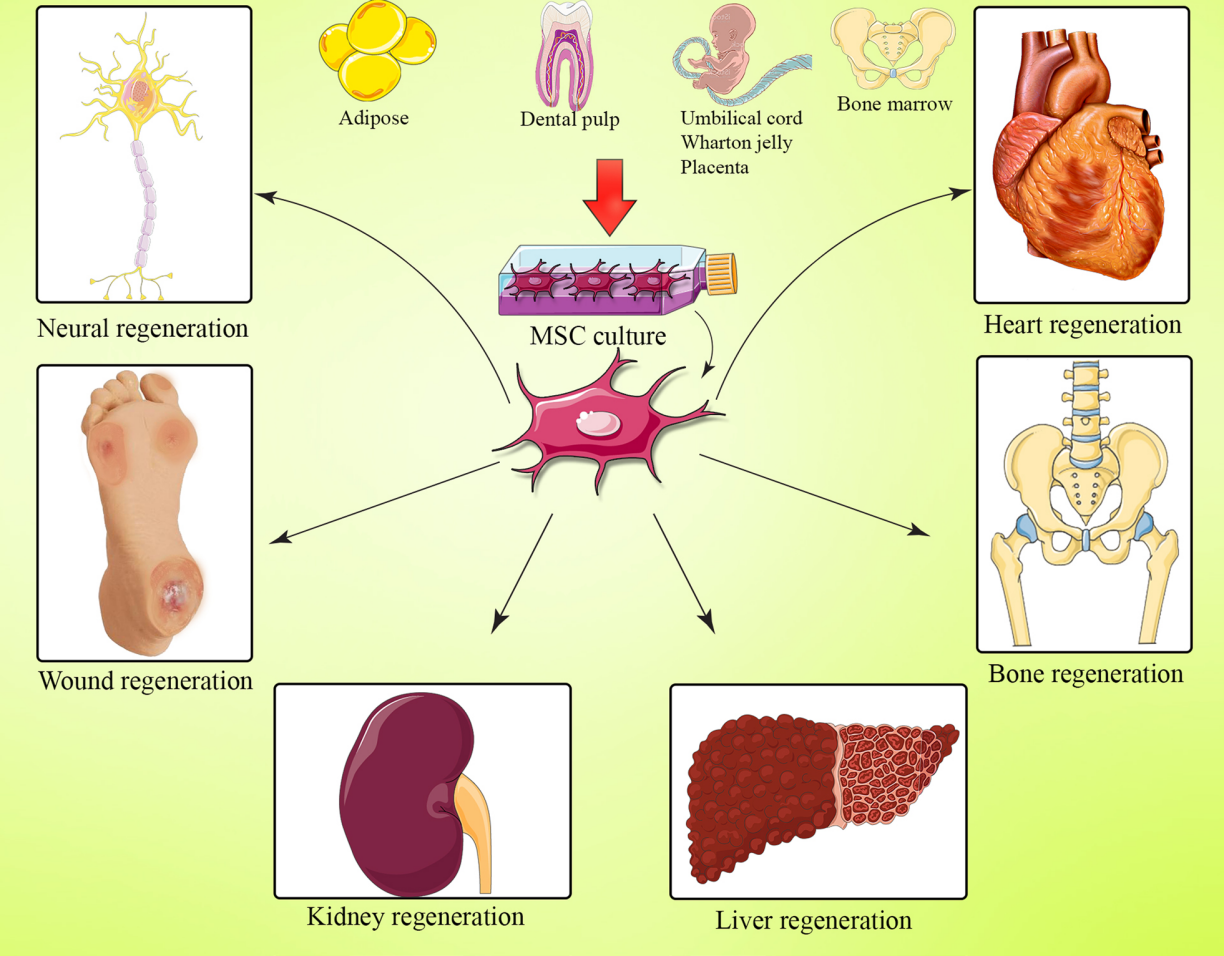
Effect of bone marrow mesenchymal stem cell-based regenerative medicine

So far, increasing data have lately studied the effects of MSCs in the treatment or regeneration of various disorders (Table 1). In this section, we reviewed the latest clinical studies that investigate the potential contribution of MSCs in the regenerative medicine, as shown in Fig. 1.

**Table 1:** Clinical application of bone marrow mesenchymal stem cells in regenerative medicine

Disease	Infusion method	Sample size	Cell mass	Cell source	Study phase	Serious adverse event	Outcome	NCT number	Reference
ALS	I.T	26	15 ± 4.5 × 10^6^ cell	BMSC	I/IIa	No	ALSFRS were significantly reduced. FVC and WSs were stable in patients	N/A	[71]
ALS	I.T	10	N/A	BMSC	I	No	MRI showed no structural changes (including tumor formation) in either the brain or the spinal cord. However, the lack of postmortem material prevents any definitive conclusion about the vitality of the MSCs after transplantation	N/A	[72]
ALS	I.T	15	10 × 10^6^ cell	BMSC	N/A	No	Reducing of the disease was indicated following MSCs therapy	N/A	[73]
ALS	I.M I.M and I.T	12 14	1 × 10^6^ cell 1, 1.5 and 2 × 10^6^ cell I.T 24, 36 and 48 × 10^6^ cell I.M	MSC-NTF	I/II IIa	No	The rate of progression of the FVC and ALSFRS was reduced. The results suggest that IT and IM administration of MSC-NTF cells in patients with ALS is safe and provide indications of possible clinical benefits	NCT01051882 NCT01777646	[74]
ALS	I.T	8	1 × 10^6^/kg	BMSC	I	No	There was no acceleration in the reduction in the ALSFRS-R, Appel ALS score, and FVC. Elevation of TGF-β and IL-10. Reduction in MCP-1	NCT01363401	[75]
ALS	I.T	27	1 × 10^7^–10^8^ cell	Adipose MSC	I	No	Elevation of CSF protein and nucleated cells along with MRI of thickened lumbosacral nerve roots	N/A	[76]
ALS	I.T	67	30 × 10^6^ cells	WJ-MSC	N/A	No	Median survival time increased twofold in patients	N/A	[77]
PD	I.A	5	N/A	BMSC	I	No	Autologous BMSCs is safe and reduce disease progression	NCT01824121	[78]
PD	I.A	5	1.2–2 × 10^6^/kg	BMSC	II	No	Participants were alive and motor function rating scales remained stable for at least 6 months during the 12-month follow-up period	NCT01824121	[79]
PD	I.T	7	1 × 10^6^/kg	BMSC	N/A	No	Improvement was found in symptoms like facial expression, gait, and freezing episodes	N/A	[80]
SCI	I.T	3	15 × 10^6^ cell	BMSC	I	No	No improvement in their sensory scores without any changes in the AIS and SCIM-III scores. No motor recovery was observed in any of the participants	N/A	[81]
SCI	I.V	13	0.84–1.6 × 10^8^ cell	BMSC	II	No	ASI, ISCSCI-92, and SCIM-III functional improvements after MSC injection	N/A	[82]
SCI	I.T	6	1.2 × 10^6^/kg	BMSC	I	No	MSCs can be safely administered through intrathecal injection in spinal cord injury patients	NCT02482194	[83]
SCI	Interalesion	14	1 × 10^7^ cell	BMSC	I	No	Improvements in tactile sensitivity and eight participants improved lower limbs motor functional gains, chiefly in the hip flexors and developments in urologic function	NCT01325103	[84]
SCI	subarachnoid	10	3 × 10^7^ cell	BMSC	II	No	Improvement in bladder compliance and active muscle reinnervation	NCT0216590	[85]
SCI	I.T	9	10 × 10^7^ cell	BMSC	II	No	Improve sensitivity, motor power, spasms, spasticity, neuropathic pain, sexual function or sphincter dysfunction in the SCI patients	NCT02570932	[86]
SCI	I.T	14	9 × 10^7^ cell	Adipose MSC	N/A	Yes	Several patients showed mild improvements in neurological function	N/A	[87]
SCI	I.T	10	10 × 10^6^ cells	WJ-MSC	I/IIa	No	Significant improvement in pinprick sensation. Increase in bladder maximum capacity	NCT03003364	[88]
Stroke	I.V	31	N/A	BMSC	N/A	No	Improvements in NIHSS score, motor-Fugl-Meyer scores, and task-related functional MRI activity in motor cortex-4a. There were no remarkable progresses in Barthel Index, NIHSS, and modified Rankin scores	NCT 00,875,654	[89]
Stroke	I.V	15 21	0.5, 1.0, and 1.5 × 10^6^/kg 1.5 × 10^6^/kg	BMSC	I II	No	Barthel Index scores increased. Electrocardiograms, laboratory tests, and computed tomography scans of chest/abdomen/pelvis suggesting that BMSCs could alleviate the stroke	NCT01297413	[90]
Stroke	Stereotactic	10	20–50 × 10^6^ cell	BMSC	I	No	Improvement in the motor function	N/A	[91]
Stroke	I.V	17	2 × 10^6^/kg	BMSC	II	No	NIHSS score, modified Rankin Scale or Barthel Index did not improve after the transplantation. There was an improvement in absolute change in median infarct volume	NCT01461720	[92]
Primary biliary cirrhosis	I.V	7	0.5 × 10^6^ /kg	UC-MSC	N/A	No	Reduction in ALP and GGT. UC-MSC therapy is feasible and well tolerated in patients with primary biliary cirrhosis	NCT01662973	[93]
Ischemic-type biliary lesions	I.V	12	1 × 10^6^/kg	UC-MSC	I	No	Reduction in ALP, GGT, and total bilirubin	NCT02223897	[94]
ACLF	I.V	9	1 × 10^6^/kg	BMSC	I/II	No	Improvement in CP, MELD, and ACLF	N/A	[95]
ACLF	I.V	110	1–10 × 10^5^/kg	BMSC	N/A	No	Improvement in serum total bilirubin, and MELD scores. Enhancing liver function and reducing the prevalence of severe infections	NCT01322906	[96]
Severe liver disease	I.A	58	0.47 ± 0.15 × 10^8^/kg	BMSC	N/A	No	Expansion of macrophages concurrent with an upregulated expression of genes involved in inflammatory and regenerative pathways. With the negative results from the clinical trial, the impact of the liver stem cell therapy has to be interpreted as weak	N/A	[97]
Alcoholic cirrhosis	I.A	72	5 × 10^7^ cell	BMSC	II	No	Reduction in the proportion of collagen. Improvement in Child–Pugh scores	NCT01875081	[98]
ARVD	I.A	39	1, 2.5 and 5.0 × 10^5^ cells/kg	Adipose MSC	Ia	No	Increase renal blood flow. Reduction in hypoxia, renal vein inflammatory cytokines, and angiogenic biomarkers	NCT02266394	[99]
ADPKD	I.V	6	2 × 10^6^/kg	BMSC	I	No	eGFR value declined and the level of serum creatinine enhanced	NCT02166489	[100]
CKD	I.V	7	1–2 × 10^6^ /kg	BMSC	I	No	Variations in eGFR and serum creatinine were not statistically significant	NCT02195323	[101]
AKI	I.V	16	250 × 10^6^ cell	BMSC	I/II	No	Stimulates an immunotherapeutic response that initiates an enhanced phenotypic alteration from tissue injury to tissue repair	NCT 03,015,623	[102]
Ischemic	Intramyocardial	60	77.5 ± 67.9 × 10^6^ cell	BMSC	II	No	Left ventricular end-systolic volume was significantly reduced; also LVEF, stroke volume, and myocardial mass remarkably improved	NCT00644410	[103]
Ischemic	Intramyocardial	14	15 × 10^7^ cell	BMSC	I	No	Quality of life was improved along with a substantial decrease in angina scores	NCT01557543	[104]
Ischemic	TESI	125	15 × 10^7^ cell	BMSC	II	No	Quality of life was significantly improved by MSCs. LEVF, left ventricular volumes, scar size, 6-min walking distance, and peak oxygen consumption did not differ significantly among groups	NCT02501811	[105]
Ischemic	I.V	13	1 × 10^6^/kg	Adipose MSC	IIa	No	No efficacy end points were remarkable between treatment groups; however, a trend toward improvement was observed in the NIHSS scores	NCT01678534	[106]
AMI	Intracoronary	116	6 × 10^6^ cell	WJ-MSC	N/A	No	Increase in the myocardial viability and perfusion within the infarcted territory. Increase in the LVEF	NCT01291329	[107]
AMI	N/A	100	100 × 10^6^ cell	BMSC	II	No	Improvement in cardiac function, induction of remodeling and regeneration, and improvement in quality of life	NCT03047772	[108]
HF	I.V	30	1 × 10^6^/kg	UC-MSC	I/II	No	Reduction in ejection fraction. Improvements in left ventricular function, functional status, and quality of life	NCT01739777	[109]
HF	N/A	8	1.2–6.5 × 10^7^ cell	BMSC	N/A	No	There were no major differences in B-type natriuretic peptide, LVEF, and peak oxygen uptake at 2 months	N/A	[110]
NIDCM	TESI	34	N/A	BMSC	I/II	No	MSC therapy improves a variety of parameters in NIDCM irrespective of patient sex	N/A	[111]
NIDCM	TESI	37	10 × 10^7^ cell	BMSC	I/II	No	Minnesota Living with Heart Failure Questionnaire score decreased. The Major Adverse Cardiac Event rate was lower in allo vs. auto. Also, TNF-α decreased, to a greater extent in allo vs. auto at 6 months	NCT01392625	[112]
HLHS	Intramyocardial	30	2.5 × 10^5^ /kg	BMSC	I/II	No	This study was determined the safety, feasibility, and usefulness of MSC administration into the left ventricular myocardium	NCT02398604	[113]
DCM	Intracoronary	53	4.9 ± 1.7 × 10^8^ cell	BMSC	N/A	No	LVEF, NYHA class, and myocardial perfusion had improved significantly in the BMSC group; however, LVEDd remained unchanged	N/A	[114]
Refractory angina	Intramyocardial	60	N/A	Adipose MSC	N/A	No	Patients receiving ASCs had improved cardiac symptoms and unchanged exercise capacity	NCT01449032	[115]
OA	Intra-articular	12	1, 10 and 50 × 10^6^ cell	BMSC	I/II	No	Improved KOOS pain, symptoms, quality of life, and WOMAC. The levels of pro-inflammatory monocytes/macrophages and IL-2 reduced in the synovial fluid after intervention	NCT02351011	[116]
OA	Subchondral	140	7800 MSCs/mL in 20 ml	BMSC	N/A	No	MSCs had a significant effect on pain to postpone or avoid the TKA in the contra lateral joint of patients with OA	N/A	[117]
OA	Intra-articular	60	5727 MSCs/mL in 40 ml	BMSC	N/A	No	Implantation of MSCs in the subchondral bone of an osteoarthritic knee is more effective to postpone TKA than injection of the same intra-articular dose in the contralateral knee	N/A	[118]
OA	Intra-articular	60	100 × 10^6^ cell	BMSC	II	No	Treatment with BMSC related to platelet-rich plasma was demonstrated to be a feasible alternative treatment for individuals with OA	NCT02365142	[119]
OA	Intra-articular	30	10 or 100 × 10^6^ cell	BMSC	I/II	No	BMSCs together with hyaluronic acid is a safe and viable process that leads to a clinical and functional improvement in knee OA	NCT02123368	[120]
OA	Intra-articular	18	N/A	BMSC	N/A	Yes	Improve the pain, function and daily living activities and quality of life subscales	N/A	[121]
OA	Intra-articular	13	61 ± 0.6 × 10^6^ cell	BMSC	I/II	No	Normalized KOOS improved significantly. Mean knee cartilage thickness measured by MRI improved significantly	NCT02118519	[122]
OA	Intra-articular	18	2, 10, 50 × 10^6^ cell	Adipose MSC	I	No	Significantly improved pain levels and function	N/A	[123]
OA	Intra-articular	30	100 × 10^6^ cell	Adipose MSC	I	No	MRI Osteoarthritis Knee Score indicated modification of disease progression and improved pain levels and function	N/A	[124]
OA	Intra-articular	12	1 × 10^8^ cells	Adipose MSC	IIb	No	Significant improvement in the WOMAC score. Provided satisfactory functional improvement and pain relief for patients	N/A	[125]
OA	Intra-articular	40	20 × 10^6^ cell	UC-MSC	I/II	No	Significantly improved pain levels and function. Pain Visual Analog scale was significantly lower in the MSC group	NCT02580695	[126]
OA	N/A	29	1 × 10^6^ cell	UC-MSC	N/A	No	Visual analog scale showed decreased pain. MSC Decreased WOMAC score	NCT03800810	[127]
Bone fracture	Percutaneous	22	50–100 × 10^6^ cell	BMSC	I/II	No	TUS, GDE score was improved and pain at palpation at the fracture site was reduced	NCT02020590	[128]
Bone fracture	Intramedullary I.V	20	4 × 10^7^ cells 2 × 10^8^ cells	WJ-MSC	I/IIa	No	VAS, ODI, and SF-36 scores significantly improved. Promoted bone architecture	N/A	[129]
Mandibular lesions	Intralesional	20	N/A	BMSC	N/A	No	Increase in bone density with respect to the baseline levels. The percent of reduction in the defects’ size was significantly higher compared with control	N/A	[130]
Wound healing	Intralesional	8	1 × 10^6^ cell	BMSC	N/A	No	Reduction in ulcer size or complete wound closure	N/A	[131]
Wound healing	N/A	316	N/A	Adipose MSC	N/A	No	Granulation tissue coverage rate and thickness of granulation tissue were significantly improved	N/A	[132]
Diabetic foot ulcers	N/A	59	1 × 10^6^ cells	Adipose MSC	N/A	No	Complete wound closure was achieved for 82% at week 12. Kaplan–Meier median times to complete closure were reduced	NCT02619877	[133]
Diabetic foot ulcers	Endovascular	53	4.8 to 8.6 × 10^7^ cell	UC-MSC	N/A	No	Significant increase in neovessels, accompanied by complete or gradual ulcer healing	N/A	[134]
Diabetic foot ulcers	N/A	114	N/A	Autologous micro-fragmented adipose tissue	N/A	No	The skin tropism was improved in the treatment group	NCT03276312	[135]
Uterine injury	Intrauterine	10	N/A	UC-MSC	I	No	The volume of the uterus, and cesarean scar diverticulum showed an improving tendency	NCT03386708	[136]
Vocal fold	Local injection	16	0.5–2 × 10^6^ cell	BMSC	I/II	No	Voice Handicap Index was meaningfully enhanced	NCT01981330	[137]

NIDCM, non-ischemic dilated cardiomyopathy; HF, heart failure; AMI, acute myocardial infarction; HLHS, hypoplastic left heart syndrome; TESI, transendocardial stem cell injection; I.V, intravenous; I.T, intrathecal; I.L, intralesional; I.A, interatrial; I.M, intramuscular

### Neural regeneration

The application of BMSCs has demonstrated promising therapeutic results in the treatment of neurological diseases. Amyotrophic lateral sclerosis (ALS), also known as motor neuron disease, is a neurodegenerative disorder that leads to degeneration of the motor neurons that causes paralysis and muscle weakness [138, 139]. Syková et al. [71] carried out a study that intrathecally injected 15 ± 4.5 × 10^6^ autologous BMSCs into 26 patients with ALS. After mesenchymal stem cells transplantation (MSCT), ALS functional rating scale (ALSFRS) significantly reduced, forced vital capacity (FVC) remained stable or above 70%, and weakness scales (WSs) were stable in 75% of patients. They have shown that the intrathecal BMSCs intervention in ALS patients is a safe method and it can slow down the development of the disease. There were no significant adverse events related to the trial during and after transplantation of BMSCs. Barczewska and colleagues indicated that three intrathecal injections of 30 × 10^6^ Wharton’s jelly-MSCs (WJ-MSCs) improved ALSFRS [77]. They showed that WJ-MSCs are safe and effective in individuals that suffer from ALS. However, one other group found that intrathecal injection of autologous adipose MSCs does not improve clinical symptoms of ALS patients [76]. Their results indicated that the levels of CSF protein and nucleated cells were increased and ALSFRS-R showed development of disease in all treated patients. In the trial by OH et al., autologous BMSCs were injected to treat seven participants that suffer from ALS [75]. The participants were injected twice with autologous BMSCs (one million cells per kg) and followed up for 12 months. No serious adverse events were reported during the follow-up period. Furthermore, during the 12-month follow-up, there was no acceleration in the decrease in the ALSFRS-Revised (ALSFRS-R) score, Appel ALS score, and FVC. Moreover, CSF analysis showed that the levels of TGF-β and IL-10 were evaluated, while MCP-1, which is chemokine-related and exacerbates the motor neuron damage in ALS, was decreased. Their results exhibited that two repeated MSC infusions have safety and feasibility for at least 1 year in seven individuals; nevertheless, the study has some limitations such as low number of participants and short-time follow-up. In another study [73], 15 ALS patients were transplanted with autologous BMSCs. These 15 patients were divided into two groups (group 1: patients who had ALS with an inherently slow course, group 2: individuals who had ALS with an inherently rapid course) and received three intrathecal infusions of MSCs. There were no significant adverse events in the course of multiple intrathecal injections of MSCs. In group 1, there were no major changes in the rate of disease development and in group 2 ameliorating of the disease was indicated following MSCs therapy. According to their observation, the response of patients with ALS to treatment with MSCs was variable. Also, the authors indicated that due to the small number of patients, less subgroups were available for statistical analysis, limiting their ability to draw conclusions from the data.

Spinal cord injury (SCI) is usually related to devastating results. The damage to the spinal cord leads to injury to the motor, sensory, and autonomic roles of the spinal cord that affects patients’ well-being such as their physical and psychological state [140, 141]. In a phase I, nonrandomized, uncontrolled study by Mendonça et al. [84], 15 SCI patients were administered 1 × 10^7^ cells/ml MSCs. The results of the investigation revealed that SCI symptoms were meaningfully decreased by MSCT, all participants showed variable improvements in tactile sensitivity, and eight participants improved lower limb motor functional gains, chiefly in the hip flexors. Seven patients revealed sacral sparing and developed American Spinal Injury Association impairment scale (AIS) grades B or C – partial damage. Nine participants had developments in urologic function and one patient showed alterations in somatosensory evoked potentials (SSEP) 3 and 6 months after MSCT. These results stated that treatment with MSCs ameliorated the organ malfunction in people with SCI and has clinical safety, because no serious adverse effects were reported. The authors indicated that their results should be confirmed in larger and controlled clinical trials. Albu and colleagues have been demonstrated that intrathecal administration of WJ-MSCs considerably improved the pinprick sensation in the dermatomes below the level of damage [88]. Further results showed that bladder maximum capacity was elevated and bladder neurogenic hyperactivity and external sphincter dyssynergy were reduced. In another study [85], ten SCI subjects received four subarachnoid injections of 30 × 10^6^ autologous BMSCs, maintained in autologous plasma, at weeks 1, 16, 28, and 40 of the trial and followed up for 12 months. There were no adverse events and all participants tolerated the therapy. Vaquero et al. [86] demonstrated that MSCT is safe and improves sensitivity, motor power, spasms, spasticity, neuropathic pain, sexual function, or sphincter dysfunction in the SCI patients. The results of their study have shown that 55.5% of patients improved in SSEP and 44.4% of patients ameliorated in voluntary muscle contraction together with intralesional active muscle reinnervation. Hur et al. carried out a study in which 14 patients with SCI were administered intrathecally 9 × 10^7^ adipose MSCs [87]. Their observations showed mild progresses in neurological function. No serious adverse events were observed. In a phase 2 study, 13 patients with SCI were intravenously administered a single dose of autologous MSCs cultured in auto-serum [82]. The results of this trial revealed that SCI symptoms were considerably declined by MSC therapy, ASI, International Standards for Neurological and Functional Classification of Spinal Cord (ISCSCI-92), and Spinal Cord Independence Measure (SCIM-III) demonstrated functional improvements after MSC injection. No severe adverse effects were related to MSC administration.

Parkinson’s disease (PD) is a neurological disorder principally characterized by the deterioration of motor activities due to the impairment of the dopaminergic nigrostriatal system [142, 143]. It has been indicated that MSCs improved the symptoms of PD. In a phase I controlled, randomized clinical study, patients that suffer from progressive supranuclear palsy were administered autologous BMSCs via intra-arterial injection [78]. The results of the study exhibited that autologous BMSCs are safe and reduce disease progression. Canesi et al. [79] have demonstrated that injection of MSCs into cerebral arteries of PD patients led to positive results in 17 PD participants: all treated participants were alive and motor function rating scales remained stable for at least 6 months during the 12-month follow-up period. One patient died 9 months after the injection for reasons not associated with cell infusion or to disease development.

In a study conducted by Jaillard and colleagues in 2019 [89], 31 individuals with subacute stroke were administered the intravenous injections of autologous BMSCs. The results of the trial exhibited significant improvements in motor-National Institute of the Health Stroke Scale (NIHSS) score, motor-Fugl-Meyer scores, and task-related functional MRI activity in motor cortex-4a. However, there was no remarkable progress in Barthel Index, NIHSS, and modified Rankin scores. In general, their results suggested that BMSCs improved motor recovery via sensorimotor neuroplasticity. In another study, 17 patients with subacute middle cerebral artery infarct received two million cells/kg autologous BMSCs [92]. During the follow-up process, NIHSS score, modified Rankin Scale or Barthel Index did not improve after the transplantation. Nonetheless, there was a significant improvement in absolute change in median infarct volume, but no treatment-related adverse effects were observed.

In sum, these outcomes suppose that BMSCs can safely and efficiently treat neural diseases, inhibit disease development, and considerably ameliorate the quality of life and clinical manifestations of patients. Consequently, BMSCs can become a new option for the clinical treatment of neural diseases.

### Liver regeneration

The potential of BMSCs to differentiate into the endodermal lineage, such as hepatocyte‐like cells, makes them an attractive alternative for the treatment of liver diseases [144]. Some clinical studies have demonstrated the efficacy and feasibility of BMSC therapy in patients with liver diseases. The effect of BMSCs has been studied in individuals suffering from liver cirrhosis by Suk et al. [98]. Seventy-two patients were enrolled in this trial and randomly classified into three groups: one control group and two autologous BMSC groups that received one-time or two-time hepatic arterial administrations of fifty million autologous BMSCs 30 days after BM aspiration. Fibrosis quantification exhibited that in one-time and two-time BMSC groups there are a reduction of 25% and 37% in the proportion of collagen, respectively. In addition, the Child–Pugh (CP) scores of both test groups were meaningfully improved following BMSC administration in comparison with the control group. No serious adverse events were associated with MSC injection during the 12-month follow-up. Wang and coworkers have found that intravenous injection of UC-MSCs (0.5 × 10^6^ cells/kg) is feasible and well tolerated in patients with primary biliary cirrhosis (PBC) [93]. They exhibited that MSCs significantly decreased the level of ALP and GGT; however, there were no considerable changes in serum AST, ALT, total bilirubin, albumin, prothrombin time activity, or immunoglobulin M levels. Similarly, Zhang et al. [94] have demonstrated that intravenous administration of 1.0 × 10^6^ cells/kg UC-MSCs is safe and efficient for patients with ischemic-type biliary lesions after liver transplantation. According to their results, MSCs therapy reduced the serum ALP, GGT, and total bilirubin. In a randomized placebo-controlled phase I–II single-center study, nine patients that suffer from acute-on-chronic liver failure (ACLF) grades 2 and 3 were enrolled [95]. The experiment group (n = 4) received standard medical therapy along with five injections of 1 × 10^6^ cells/kg of BMSC for 3 weeks. There were no transplant-related adverse events; however, one patient in the experiment group showed hypernatremia and a gastric ulcer, after the third and fifth administrations, respectively. Furthermore, MSCT revealed a considerable improvement in CP, model for end-stage liver disease (MELD), and ACLF (grade 3 to 0). Thus, MSCT is safe and viable in individuals with ACLF. In an open-label non-blinded randomized controlled study conducted by Lin et al. [96], 110 patients with hepatitis B virus (HBV)-related ACLF were enrolled in this trial. These patients were divided into two groups: control group (N = 54) was treated with standard medical therapy only and the intervention group (N = 56) was injected four times with 1.0–10 × 10^5^ cells/kg allogeneic BMSCs, and then followed up for 6 months. There were no serious adverse events associated with transplantation. The results of that study demonstrated that MSCT significantly improved clinical laboratory measurements, such as serum total bilirubin, and MELD scores in comparison with control group. In addition, mortality from multiple organ failure and prevalence rate of serious infection in the intervention group was lower than that in the control group. Their results clearly established the safety and feasibility of the clinical use of peripheral administration of allogeneic BMSCs for subjects with HBV-associated ACLF, and markedly enhanced the survival rate through enhancing liver function and reducing the prevalence of severe infections.

In summary, MSCT can meaningfully ameliorate the clinical manifestations of these patients, reduce the liver fibrosis, and inhibit the development of disease.

### Kidney regeneration

Hurt to renal cells can occur because of a wide range of ischemic and toxic insults and results in inflammation and cell death, which can lead to kidney damage. Inflammation has a significant role in the damage of renal cells, as well as following cellular regeneration processes [3, 145]. Various investigations have consistently demonstrated a supportive effect of MSC on acute and chronic renal injury [146]. Makhlough et al. declared that intravenous administration of 1–2 × 10^6^ cells/kg into seven patients with chronic kidney disease failed to induce remission [101]. They indicated that variations in estimated glomerular filtration rate (eGFR) and serum creatinine during the 18-month follow-up were not statistically significant. Nonetheless, no severe adverse events were reported, and they could not assess the efficacy because of their study design. Authors postulated that limited sample size and lack of a control group led to the lack of success. A study conducted by Swaminathan et al. in 2021, has displayed the effect of allogeneic BMSCs in acute kidney injury patients. They have shown that treatment of MSCs with SBI-101 stimulated an immunotherapeutic response that initiated an enhanced phenotypic alteration from tissue injury to tissue repair [102]. In a single-arm phase I clinical trial carried out by Makhlough et al. [100], six patients with autosomal dominant polycystic kidney disease (ADPKD) were intravenously injected 2 × 10^6^ cells/kg autologous BMSCs. The results of the study showed that the mean eGFR value declined and the level of serum creatinine enhanced during the 1-year follow-up. Moreover, no remarkable modifications in renal function parameters and blood pressure were observed during the year after intervention. However, there were no severe adverse events after 1-year follow-up. In addition, the authors indicated that there are some reasons for the lack of success, including small number of patients, absence of a comparison group, limited follow-up period, single dose administration, and they did not utilize htTKV as a surrogate endpoint. Abumoawad and colleagues have established that adipose MSCs enhanced blood flow, GFR and reduced inflammatory injury in poststenotic kidneys of individuals that suffer from atherosclerotic renovascular disease (ARVD) [99]. Their results illustrated that mean renal blood flow was considerably enhanced, and hypoxia, renal vein inflammatory cytokines, and angiogenic factors were considerably attenuated.

### Heart regeneration

Heart disease is the first and most frequently diagnosed disease and the leading cause of disease death [147]. When cardiomyocytes are damaged via ischemic and other factors, the remaining viable cardiomyocytes have a restricted ability to proliferate and dead cardiomyocytes are changed by non-contractile fibrous tissue, leading to functional impairment that elicits the progression of heart failure. According to the developing number of patients with heart disease, there is a vital need to expand an innovative remedy to rescue deteriorating hearts. Regenerative medicine and cell therapy are the upcoming therapeutic opportunities for heart diseases. According to the literature, the transplantation of BM-derived cells and cardiac stem cells into deteriorating hearts appeared to provide functional benefits [148, 149].

In a study by Yagyu et al. [110], 8 individuals with symptomatic heart failure were infused with BMSCs. During the follow-up period, no serious adverse events were observed. There were no major differences in B-type natriuretic peptide, left ventricular ejection fraction (LVEF), and peak oxygen uptake at 2 months. The results of this study recommend further research regarding the feasibility and efficacy of MSCs. In a study by Gao et al. [107], 116 patients with acute myocardial infarction randomly received an intracoronary injection of WJ-MSCs. They indicated that MSCs therapy elevated the myocardial viability and perfusion within the infarcted territory. In addition, the LVEF was elevated and LV end-systolic volumes and end-diastolic volumes were decreased in the WJ-MSCs group.

Chan et al. demonstrated that intramyocardial infusion of autologous BMSCs in conjunction with transmyocardial revascularization or coronary artery bypass graft surgery was technically feasible and could be performed safely. The results showed that regional contractility in the cell-treated regions improved during the 1-year follow-up; also, the quality of life was improved along with a substantial decrease in angina scores at 12 month post-treatment [104]. In a study by Kaushal et al. [113], 12 participants with hypoplastic left heart syndrome were transplanted with allogeneic human MSCs (2.5 × 10^5^ cells/kg). This study determined the safety, feasibility, and usefulness of MSC administration into the left ventricular myocardium. No serious adverse effects were reported during the trial. Mathiasen et al. observed that after BM-MSCT, left ventricular end-systolic volume was significantly reduced, also LVEF, stroke volume, and myocardial mass remarkably improved [103]. In addition, a major decrease in the amount of scar tissue and quality of life score was observed. No side effects were identified. In a randomized, double-blind, placebo-controlled, multicenter, phase II study, 100 patients with anterior ST elevation myocardial infarction received autologous BMSCs and atorvastatin (ATV) treatment. The results of that study represented the absolute change of LEVF within 12 months, improvement in cardiac function, induction of remodeling and regeneration, and improvement in quality of life [108]. Recently, Celis-Ruiz and coworkers conducted a study in which intravenous administration of adipose MSCs within the first 2 weeks of ischemic stroke onset is safe at 24 months of follow-up [106]. In a study conducted by Hare et al. [112], 37 non-ischemic dilated cardiomyopathy patients were divided into two groups and received 10 × 10^7^ allogeneic and autologous BMSCs. Minnesota Living with Heart Failure Questionnaire score decreased in both groups. The major adverse cardiac event rate was lower in allo vs. auto. Also, TNF-α decreased, to a greater extent in allo vs. auto at 6 months. These results suggested the clinically meaningful efficacy of allogeneic vs. autologous BMSCs in non-ischemic dilated cardiomyopathy patients. Qayyum et al. have found that intra‑myocardial injections of autologous adipose MSCs ameliorated cardiac functions and unchanged exercise capacity, in contrast to deterioration in the placebo group [115].

Levy et al. indicated that after allogeneic BMSCs in patients with chronic stroke, Barthel Index scores increased. Moreover, electrocardiograms, laboratory tests, and computed tomography scans of chest/abdomen/pelvis suggest that BMSCs could alleviate the clinical symptoms in patients with stroke [90].

In sum, BMSC therapy can be an effective, achievable, and safe process that remarkably improves cardiac function and promotes patients’ quality of life.

### Bone regeneration

Bone regeneration is a hot topic of research in clinical studies. Bone regeneration is a crucial problem in numerous cases, including bone fracture, defect, osteoarthritis, and osteoporosis, which should be resolved [150–152]. Autogenous bone grafts are considered the standard approach for bone formation by means of the participants’ own cells that stimulate osteoinductive, bone conductivity, and histocompatibility in bone diseases [153]. Nevertheless, there are some shortcomings of this procedure such as unpredictable absorption, extended recovery time, and patients commonly experience pain and nerve injury at the harvest area [154–156]. With the development of understanding bone tissue biology as well as recent approaches in the improvement in tissue regeneration, the application of MSC has become an attractive subject in augmenting bone tissue forming [157, 158].

In a pilot study by Jayankura and coworkers, allogeneic BMSCs were applied to treat 22 participants with bone fractures [128]. All participants received percutaneous implantation of autologous BMSCs (5 to 10 × 10^7^ cells) into the fracture area. After intervention, Tomographic Union Score (TUS) and Global Disease Evaluation (GDE) score were improved, and pain at palpation at the fracture site was reduced. In addition, the ratio of blood samples comprising donor-specific anti-HLA antibodies enhanced at 6 months post-intervention. Three serious cell-related adverse events were reported. In another study by Shim and coworkers [129], intramedullary (4 × 10^7^ cells) and intravenous (2 × 10^8^ cells) infusion of WJ-MSCs in combination with teriparatide showed beneficial results in individuals with osteoporotic vertebral compression fractures. Their observation displayed that the mean visual analog scale, Oswestry Disability Index, and Short Form-36 scores meaningfully improved. They stated that WJ-MSCs in combination with teriparatide are viable and have a clinical profit for fracture healing by stimulating bone architecture.

Several studies investigated the effect of BMSCs in osteoarthritis (OA) patients. Chahal et al. carried out a clinical phase I/IIa trial that involved 12 individuals with late-stage Kellgren–Lawrence knee OA. These 12 patients were injected with a single intra-articular of 1 × 10^6^, 10 × 10^6^, and 50 × 10^6^ BMSCs. The results showed that patients had improved Knee Injury and Osteoarthritis Outcome Score (KOOS) pain, symptoms, quality of life, and Western Ontario and McMaster Universities Osteoarthritis Index (WOMAC) stiffness relative to baseline. Moreover, cartilage catabolic biomarkers and MRI synovitis were meaningfully lower at higher doses and the levels of pro-inflammatory monocytes/macrophages and IL-2 reduced in the synovial fluid after intervention. No serious events had occurred [116]. Dilogo et al. have reported that UC-MSCs (10 × 10^6^ cells) significantly decreased the WOMAC and could be a potentially new regenerative treatment for patients with knee OA [127]. In a study conducted by Hernigou et al. [117], 140 patients with OA received a subchondral infusion of BMSCs on one side and received total knee arthroplasty (TKA) on the contralateral knee. They demonstrated that subchondral MSCs had a significant effect on pain to postpone or avoid the TKA in the contralateral joint of patients with OA. In a phase II multicenter randomized controlled clinical trial, 60 OA patients received 10 × 10^7^ cells of autologous BMSCs along with platelet-rich plasma and followed up for 12 months [119]. No serious adverse effects were observed after MSCs injection or during follow‑up. According to the observations, treatment with BMSC related to platelet-rich plasma was demonstrated to be a feasible alternative treatment for individuals with OA, along with clinical development at the end of follow-up. Similarly, Bastos et al. have reported that MSCs alone or in combination with platelet-rich plasma are safe and have an advantageous effect on symptoms in OA individuals [121]. They found that MSCs group and MSCs + platelet-rich plasma group can improve the pain, function and daily living activities, and quality of life subscales. Ten adverse events were reported in three participants in the MSCs group and in two of the MSCs + platelet-rich plasma group. PERS and colleagues reported another clinical phase Ia study that involved 19 individuals suffering from knee OA [123]. These 18 individuals were classified into three groups and received a single intra-articular administration of 2 × 10^6^, 10 × 10^6^, and 50 × 10^6^ adipose MSCs. According to their results, individuals had experienced significant improvement in pain levels and function. There were no severe adverse events; however, 4 individuals experienced transient knee joint pain and swelling after local administration. In a long-term follow-up of a multicenter randomized controlled clinical trial by Espinosa et al. [120], 30 OA patients were administered the intra-articular infusion of two diverse doses of autologous BMSCs cells (10 × 10^6^ or 10 × 10^7^) versus hyaluronic acid in the treatment of OA. No adverse effects occurred after MSCT or during the 4-year follow‑up. Their results showed that intra-articular infusion of BMSCs together with hyaluronic acid is a safe and viable process that leads to a clinical and functional improvement in knee OA.

Overall, these data display that BMSCs can be a promising, safe and effective alternative for bone regeneration, significantly improve the clinical manifestation of patients, and inhibit development of diseases.

### Wound regeneration

The skin has several layers along with different compounds and roles that work together to support internal organs and serve various biological roles. It has three main layers, the epidermis, the dermis, and the subcutaneous layer [159]. Generally, skin wound healing, triggered by tissue injury, includes four stages: hemostasis, inflammation, proliferation, and maturation. MSCs can assist in all stages of the wound healing process. The use of MSCs for the treatment of skin can improve the regeneration of skin and reduce scarring. MSCs exert their functions through migration into the skin damage site, suppressing inflammation, and increasing the growth and differentiation ability of fibroblasts, epidermal cells, and endothelial cells [160, 161]. As MSCs have exhibited wound healing in many preclinical studies, the application of MSCs for chronic wounds contributes to progress toward clinical trials. Falanga et al. have demonstrated that autologous BMSCs are an impressive and safe treatment method for wound healing [131]. The results of the study indicated a trend toward a reduction in ulcer size or complete wound closure by 4–5 months. No adverse events were noted. In a study by Zhou et al., 346 patients with skin wounds were administered adipose MSCs [132]. There were no adverse events during the trial. They reported that the granulation tissue coverage rate and thickness of granulation tissue were considerably ameliorated. In an open-label phase I/II study, sixteen participants with vocal fold scarring were administered a single dose of 0.5–2 × 10^6^ cells autologous MSCs [137]. Video ratings of vocal fold vibrations and digitized analysis of high-speed laryngoscopy and phonation pressure threshold were considerably enhanced for 62–75% of the participants. Voice Handicap Index was meaningfully enhanced in eight participants, with the remaining experiencing no remarkable alteration. No serious adverse events or minor side effects were reported. Lonardi et al. observed that micro-fragmented adipose tissue improved skin tropism in patients with diabetic foot ulcer [135]. Furthermore, the results of studies have shown that adipose-derived stem cells had a beneficial effect on the full-thickness foot dorsal skin wound in diabetic mice with a considerably decreased ulcer area [162]. Recently, Huang et al. carried out a clinical study in which six subjects with intrauterine adhesion and four with cesarean scar diverticulum enrolled in this trial [136]. They found that intrauterine injection of UC-MSCs improved the endometrial thickness, cesarean scar diverticulum, and the volume of the uterus.

## Conclusion

In the last decades, optimizations of isolation, culture, and differentiation procedures have permitted MSCs to improve closer to clinical uses for improving disorders and various tissue regeneration. MSCs have some important characteristics that make them preferred candidates to use for regenerative medicine: immunomodulatory capability valuable to improve immune system abnormalities, paracrine or autocrine roles that produce growth factors, and the vital potential to differentiate into various cells. Several clinical trials have reported that both autologous and allogeneic MSCs are valuable sources for tissue forming. Particularly, autologous MSCs signify the chief sources examined safe for administration and minimization of immunological threat, regardless of the lack of reported grievances concerning allogeneic MSC-based therapy. According to the studies described in this literature, administration of MSCs appear to be more effective and the usefulness of MSC therapy in bone and heart disorders has been broadly established. In terms of safety, no significant relationship was found between the MSC therapy and incidence of cancer and infection. Intravenous injection of MSCs is the most widely used form of administration and the dosage commonly fluctuates between 1 × 10^6^ cells/kg and 2 × 10^8^ cells/kg. According to the literature works mentioned in this review, the repeated administration of MSCs suggests being more beneficial than a single injection. In addition, the effectiveness of MSCs therapy in osteoarthritis disorder has been widely established. Long-term follow-up studies exhibited that serum tumor markers did not enhance before and 3 years after MSCs therapy. Nevertheless, there is still a lack of reliable scientific data on the mechanisms whereby the MSC therapy improves the numerous disorders that can develop the MSC modification and increase their prospective clinical application.

## Data Availability

Not applicable.

## Abbreviations

**ALS**: Amyotrophic lateral sclerosis
**AIS**: Association impairment scale
**ALSFRS**: ALS functional rating scale
**ALSFRS-R**: ALSFRS-revised
**ACLF**: Acute-on-chronic liver failure
**ADPKD**: Autosomal dominant polycystic kidney disease
**ATV**: Atorvastatin
**BM**: Bone marrow
**BMSCs**: Bone marrow mesenchymal stem cells
**COX2**: Cyclooxygenase 2
**CP**: Child–Pugh
**DCs**: Dendritic cells
**ESCs**: Embryonic stem cells
**eGFR**: Estimated glomerular filtration rate
**FVC**: Forced vital capacity
**GDE**: Global Disease Evaluation
**HSCs**: Hematopoietic stem cells
**HBV**: Hepatitis B virus
**HGF**: Hepatocyte growth factor
**IPSCs**: Induced pluripotent stem cells
**IDO**: Indoleamine 2,3-dioxygenase
**ICOSL**: Inducible co-stimulator ligands
**ISCSCI-92**: International Standards for Neurological and Functional Classification of Spinal Cord
**KOOS**: Knee injury and osteoarthritis outcome score
**LVEF**: Left ventricular ejection fraction
**MiRNAs**: MicroRNAs
MSCs: Mesenchymal stem cells
MSCT: Mesenchymal stem cells transplantation
MELD: Model for end-stage liver disease
NO: Nitric oxide
OA: Osteoarthritis
PSCs: Pluripotent stem cells
PGE2: Prostaglandin E2
SCI: Spinal cord injury
SSEP: Somatosensory evoked potentials
SCIM-III: Spinal cord independence measure
Tregs: Regulatory T cells
TUS: Tomographic Union Score
TKA: Total knee arthroplasty
WSs: Weakness scales
WOMAC: Western Ontario and McMaster Universities Osteoarthritis Index
